# Tissue-resident, memory CD8^+^ T cells are effective in clearing intestinal *Eimeria falciformis* reinfection in mice

**DOI:** 10.3389/fimmu.2023.1128637

**Published:** 2023-02-14

**Authors:** Fangyun Shi, Sixin Zhang, Ning Zhang, Ying Yu, Pei Sun, Xinming Tang, Xianyong Liu, Xun Suo

**Affiliations:** ^1^ National Key Laboratory of Veterinary Public Health Security, Beijing, China; ^2^ Key Laboratory of Animal Epidemiology and Zoonosis of Ministry of Agriculture, Beijing, China; ^3^ National Animal Protozoa Laboratory & College of Veterinary Medicine, China Agricultural University, Beijing, China; ^4^ Key Laboratory of Animal Biosafety Risk Prevention and Control (North) of MARA, Institute of Animal Science, Chinese Academy of Agricultural Sciences, Beijing, China

**Keywords:** Eimeria falciformis, resident memory T cell, intestinal immunity, Apicomplexa pathogen, protective immunity

## Abstract

*Eimeria*, a cousin of malarial parasites, causes coccidiosis that results in huge losses in the poultry industry. Although live coccidiosis vaccines have been developed and used widely for the successful control of the disease, the mechanism underlying protective immunity remains largely unknown. Using *Eimeria falciformis* as a model parasite, we observed that tissue-resident memory CD8^+^ T (Trm) cells accumulated in cecal lamina propria following *E. falciformis* infection in mice, especially after reinfection. In convalescent mice challenged with a second infection, *E. falciformis* burden diminished within 48-72 h. Deep-sequencing revealed that CD8^+^ Trm cells were characterized by rapid up-regulation of effector genes encoding pro-inflammatory cytokines and cytotoxic effector molecules. While FTY720 (Fingolimod) treatment prevented the trafficking of CD8^+^ T cells in peripheral circulation and exacerbated primary *E. falciformis* infection, such treatment had no impact on the expansion of CD8^+^ Trm cells in convalescent mice receiving secondary infection. Adoptive transfer of cecal CD8^+^ Trm cells conferred immune protection in naïve mice, indicating that these cells provide direct and effective protection against infection. Overall, our findings not only explain a protective mechanism of live oocyst-based anti-*Eimeria* vaccines but also provide a valuable correlate for assessing vaccines against other protozoan diseases.

## Introduction

The phylum Apicomplexa is home to a large number of intracellular parasites, including *Plasmodium*, *Toxoplasma gondii*, *Neospora*, *Babesia, Theileria* and *Eimeria* that are of broad medical and veterinary importance (1). Many parasitic protozoan diseases continue to rank among the world’s leading global health concerns. Among *Eimeria* parasites that are often found in a wide range of domestic animals and wildlife, *Eimeria falciformis* is a tissue-specific intracellular pathogen that infects epithelial cells in the murine cecum and proximal colon (2). Its life cycle is divided into several stages: (i) oral ingestion of sporulated oocysts, with each sporulated oocyst releasing 8 infectious sporozoites, (ii) invasion into enterocytes, (iii) 3-4 generations of schizogony (asexual reproduction), and (iv) sexual reproduction. Each infection leads to fecal discharge of a large number of oocysts, usually on days 7-13 and followed by a self-limiting that is rarely seen in natural infection with other Apicomplexa parasites like *Plasmodium sp* and *Toxoplasma gondii.* As such, *Eimeria falciformis* is an excellent model organism for studying immune memory. In previous studies, strong immune responses induced by primary *E. falciformis* infection have been shown to help establish long-term protection against subsequent infections (3), and the underlying mechanisms for lasting immunity offer a unique angle for studying the intestinal immune barrier.

As seen with many intracellular pathogens, including bacteria, viruses and parasites, resistance to *Eimeria* infection relies largely on cell-mediated immunity (CMI) (4), and the same applies to coccidiosis vaccination. Conceivably, CMI memory is capable of provoking rapid and robust responses upon re-exposure to the same pathogen, which is the basis of many vaccination protocols (5) intended for the generation of long-lasting immune memories (6). For the three main subsets of memory T-cells, i.e., central memory (Tcm), effector memory (Tem) and tissue-resident memory (Trm) cells (7), Trm cells have a tendency to stay within the site of initial infection (8, 9) and provide a first line of rapid defense, often within hours of reinfection (10). Thus, the purpose of this study was to determine if CD8^+^ Trm cells accumulate in the intestine at the time of *E. falciformis* infection and continue to confer protective immunity in secondary infection. The overall findings demonstrated that CD8^+^ Trm cells induced by primary *E. falciformis* infection are indeed the driving force for immune protection during reinfection, and our findings explained a protective mechanism of live-oocyst based anti-*Eimeria* vaccines.

## Materials and methods

### Ethics statement

All experimental procedures were approved in strict accordance with the China Agricultural University Institutional Animal Care and Use Committee guidelines (AW122022-1-1) and followed the International Guiding Principles for Biomedical Research Involving Animals.

### Mice and parasites

The life cycle of *E. falciformis* was maintained by continuous passage in Balb/c mice. 7-14 weeks-old female Balb/c mice came from Beijing Huafukang Biotechnology Co., LTD. Mice in the primary infection (PI) group were infected with 5000 *E. falciformis* at 11 weeks of age. Mice in the secondary infection (SI) group received 100 oocysts of *E. falciformis* at 8 weeks of age and reinfected with 5000 *E. falciformis* 3 weeks after the primary infection. In experiments designed for monitoring parasite development, mice at 7 weeks and 10 weeks of ages were infected twice (100 and 5000 GFP-tagged *E. falciformis*, respectively), and then reinfected with a high-dose of GFP-tagged *E. falciformis* 3 weeks later. For digital imaging, tissue samples were collected at 8 h (inoculated with 10^6^ GFP-tagged *E. falciformis*), 24 h (5 x 10^5^), 48 h (105) and 72 h (105) following reinfections in order to visualize parasites within early stages of infection. For quantification of oocyst output, feces were collected daily during patency, soaked in water, homogenized and saturated sodium chloride solution before oocysts were counted in MacMaster chambers.

### FTY720 treatment

FTY720 (Adooq Bioscience) was used to block circulatory lymphocyte (11). FTY720 was intraperitoneally injected into mice at a concentration of 1 mg/kg/d for a period of 5 days before primary and secondary infection and until the end of experiments.

### Histology and immunohistochemistry

Fresh cecal tissue was fixed with 4% paraformaldehyde (Beyotime) and then paraffin-embedded, sectioned, and stained with hematoxylin and eosin for histologic evaluation. For multi-parameter immunohistochemistry, tissues were dissected into 4 μm sections. Slides were de-paraffinized and re-hydrated. Antigen retrieval was achieved by boiling the samples in Tris EDTA (pH 9.0) in a pressure cooker. They were then blocked (10% goat serum). Anti-mouse CD4 (CST), CD8 (CST), CD69 (Abcam), CD103 (Abcam) were used as primary antibodies. These were incubated overnight at 4°C or 2 h at room temperature. Five-color multiplex fluorescence immunohistochemical staining kit (anti-rabbit secondary antibody) (Absin) was used following manufacturers’ instructions. Images were acquired using respective filters of a Nikon A1 confocal laser-scanning microscopy (Nikon), and overlaid to generate a bicolor image. For frozen sectioning, cecum tissues were incubated in a 4% methanol-free formaldehyde solution (Beyotime), then equilibrated with phosphate-buffered saline containing 20% sucrose and frozen in OCT compound (SAKURA). Each 6 μm cryosection was air-dried, washed with phosphate buffered saline, stained for DAPI (Solarbio) and mounted with ProLong Gold antifade reagent (Invitrogen). The processed tissue sections were analyzed with Olympus IX71 inverted fluorescence microscope (Olympus).

### Dissection of LPL and IEL from cecum tissues

Cecum samples were first freed from residual fat tissue, Peyer’s patches, feces and then cut into smaller pieces and incubated in Hanks’ Balanced Salt Solution with 2% FCS, 5 mM of EDTA and 2 mM of dithiothreitol for 30 min at 37°C and vortexed. The inter-epithelial lymphocytes (IEL) fraction was dissected by filtering over a 70 μm cell strainer. To recover the lamina propria lymphocytes (LPL) fraction, IEL-depleted intestine pieces were washed in Hanks’ Balanced Salt Solution supplemented with 2% FCS and enzymatically digested for 45 min at 37°C with Collagenase type IV (Solarbio), Neutral protease and DNase I (Solarbio) in 1640 medium. Single-cell suspensions were generated by filtering over a 70-μm cell strainer. The IELs and LPLs were purified by density centrifugation on a 67% and 44% percoll gradient (Cytiva).

### Flow cytometry

IELs and LPLs were passed through a 40-um cell strainer to a obtain single-cell suspension. A single-cell suspension was stimulated with PMA (50 ng/ml) and ionomycin (500 ng/ml) and incubated with brefeldin A (5 ug/ml) for 5 h, followed by staining for intracellular cytokines and surface markers. Exclusion of dead cells was performed with LIVE/DEAD Fixable Zombie Dead Cell Stain Kit (BioLegend). All cell preparations were Fc-blocked by CD16/32 antibody (BioLegend) prior to staining. Cell surface staining was performed with PerCP/Cy5.5 anti-mouse CD8α, FITC anti-mouse CD3, BV510 anti-mouse CD4, APC anti-mouse CD103, PE anti-mouse CD69 antibody and BV421 anti-mouse CD62L antibody (all from BioLegend). For detection of intracellular cytokines, cells were fixed in 4% PFA and permeabilized with BD perm/wash™ (BD Biosciences), followed by staining with Bv421 anti-mouse TNF-αand AF647 anti- mouse IFN-γ (BioLegend). Flow cytometric analysis was performed on an LSR Fortessa, and cell results were acquired using Diva software (BD Biosciences) and analyzed with FlowJo software. Sorting was performed on an Aria SORP high-speed cell sorter (BD Biosciences).

### Bulk RNA-sequencing and analysis

Three biological replicates of each cell population were sequenced with SMART Seq2. In brief, 3000 cells of each sample were directly sorted into 100 μl TrizoL. Total RNA was extracted using the standard TRIzol protocol and used for library preparation and sequencing. The library preparations were sequenced on the Illumina Novaseq™ 6000 platform by Lianchuan Biotechnology Company (Hangzhou). After obtaining sequencing reads, quality control analysis was performed for the raw data. The clean reads were mapped to the *Mus musculus* (mouse, GRCm38 assembly) reference genome sequence. Differential analysis of gene expression among naïve CD8^+^ T and CD8^+^ Trm cells were done using DESeq2. Drawings for correlation coefficient, volcano plot and heatmap were based on the normalized gene expression data. The volcano plot and heatmap were generated with the omicstudio online tools (https://www.omicstudio.cn/index).

### Adoptive transfer of MACS-sorted CD8^+^ Trm cells

Cecum CD8^+^ Trm cells were pooled from 18 reinfected mice and prepared as described above, followed by cell separation over continuous 44% and 67% Percoll (Cytiva). Cells were pressed through a 40 μm cell strainer to obtain a single cell suspension. CD8^+^ Trm cells were sorted by flow cytometer (MoFlo). A total of 1×10^6^ CD8^+^Trm cells was transferred i.v. 1 d before challenge. PBS-injected mice served as controls.

### Statistical analysis

All statistical analyses were performed using Prism 8 (GraphPad) software, with summary statistics for means ± SD and *p* values (determined by two-tailed Student’s t-test). Two levels of statistical significance were flagged by * (*p* < 0.05) or ** (*p <*0.001).

## Results

### Protective immunity induced by primary *E. falciformis* infection targeted the early schizont stage

**Figure 1:**
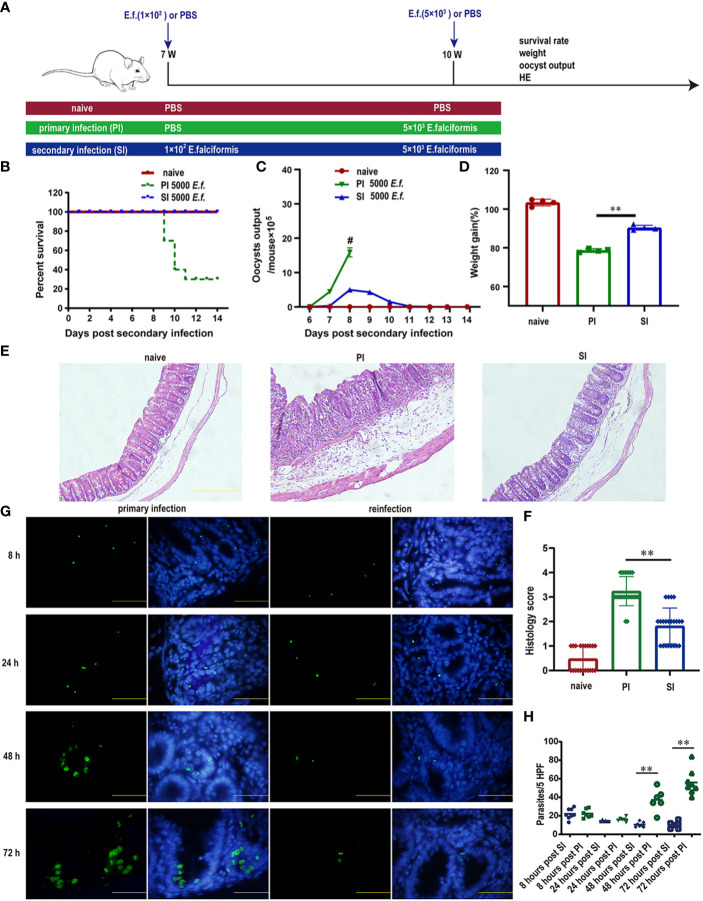
Protective immunity induced by primary *E. falciformis* (*E.f.*) infection targeted early schizonts. **(A)** Experimental design for *E. falciformis* primary infection and reinfection. Naïve mice remained uninfected for the duration of study. For the infected mice, each infection doses is indicated. **(B)** Survival curves after primary infection and secondary infection with 5000 oocysts of *E. falciformis*. *n*=9 per treatment group. **(C)** Kinetics of oocyst output at 6-13 days post primary infection and reinfection with 5000 oocysts of *E. falciformis*, # denotes the mice with severe diarrhea, to the extent that faeces collection was not possible. *n*=6 per treatment group. **(D)** Body weight change at 8 days post primary infection and secondary infection with 5000 *E. falciformis*. *n*=4 per treatment group. **(E)** H&E staining of cecum from mice at 5 days after primary infection and reinfection with 5000 *E. falciformis*. *n*=3 per treatment group, magnification ×200. **(F)** Histology scoring of cecum at 5 days after primary infection and reinfection with 5000 *E. falciformis*. **(G)** Frozen sections for *E. falciformis* (green) and DAPI (blue) in cecum from single infected and reinfected mice (to visualize parasites in the cecum at 8 and 24 h post infection, mice were infected twice with *E. falciformis* expressing GFP and reinfected after 3 weeks. *n*=3 per group, magnification ×1000. **(H)** Quantification the *E. falciformis* in cecum at 8, 24, 48 and 72 hours post primary infection and reinfection. *n*=3 per group, HPF, high power field (×1000). Bar graphs capture mean ± SD from three independent replicates, **p ≤ 0.01, PI, primary infection; SI, secondary infection.

To assess the impact of primary *E. falciformis* infection (at low doses) on resistance to reinfection, we divided Balb/c mice into three comparison groups: no infection (naïve) for the duration of the study, primary infection only (PI) and primary infection plus secondary infection (SI) with high-dose of parasites at three weeks after the primary infection (**Figure 1A**). We then measured mortality, fecal oocyst shedding, body weight as well as pathological features in these mice. Only 22% of mice infected with high-dose infection (5000 oocysts each) for the first time survived to 14 days after infection, while all SI mice receiving the same high dose survived (**Figure 1B**). SI mice produced a small amount oocysts during the patent period (**Figure 1C**). The body weight of both PI and SI mice decreased at day 8 day after high dose infection when compared with the naïve mice, but less weight loss was observed in SI than PI mice (**Figure 1D**). Histological examination revealed that pathological features in the cecum differed starkly between PI and SI mice. For example, at day 5 after high dose infection, intestinal goblet cells in SI mice were mostly intact but were almost totally lost in the PI group. Likewise, the intestinal villi in SI mice had fewer lesions than in PI mice (**Figures 1E, F**).

In the SI group, infection with GFP-tagged *E. falciformis* parasites had a relatively normal stage of sporozoites that managed to invade villous epithelial cells and migrated to crypt epithelial cells within 8-24 h after infection, as the observed amounts of sporozoites in PI and SI mice were almost identical within 24 h after infection. While the sporozoites developed into mature first-generation schizont in PI mice after 48-72 h, SI mice had degenerated first-generation schizont in crypt epithelial cells of reinfected mice (**Figure 1G**). The number of parasites in the primary infected group greatly exceeded that in the reinfected group at 48-72 hours post infection (**Figure 1H**).

### CD8^+^ T cells rose rapidly in cecal lamina propria after *E. falciformis* reinfection

**Figure 2:**
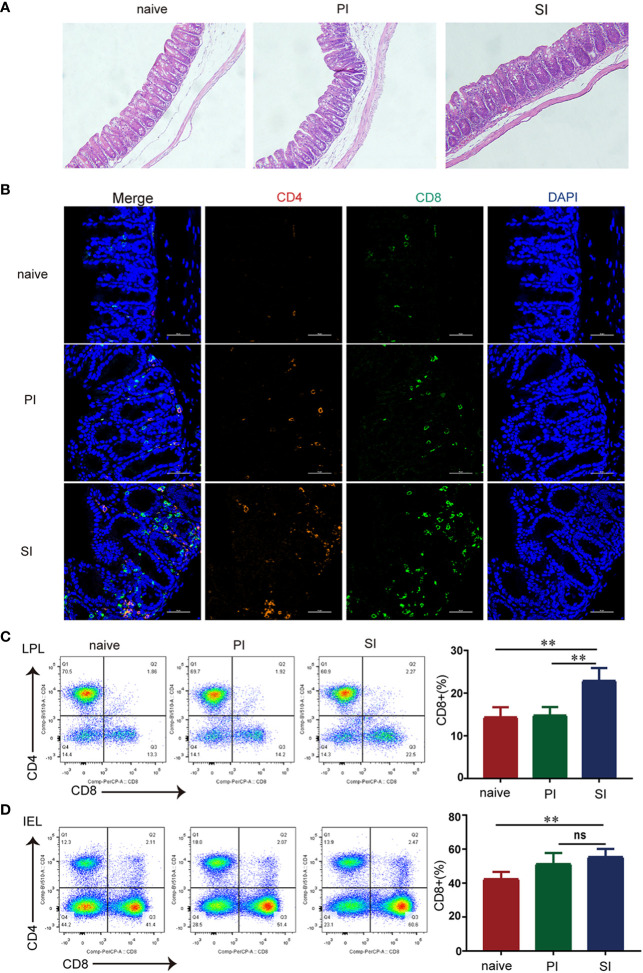
CD8^+^ T cells rose rapidly in the lamina propria of cecum after reinfection with *E. falciformis*. **(A)** H&E staining of cecum at 24 hours post primary infection and secondary infection with 5000 *E. falciformis*. *n*=3 per treatment group, magnification ×200. **(B)** Multi-parameter immunohistochemical staining of cecal CD4^+^ and CD8^+^ T-cells in three groups after 24 h primary infection and reinfection. *n*=3 per treatment group, magnification ×600. **(C)** Flow cytometric for expression of CD4*^+^* and CD8*^+^* on CD3*^+^* T cells in IEL of cecum at 24 hours after primary infection and reinfection with 5000 *E. falciformis*. *n*=6 per treatment group. **(D)** Flow cytometric for expression of CD4*^+^* and CD8*^+^* on CD3*^+^* T cells in LPL of cecum at 24 hours after primary infection and reinfection. *n*=6 per treatment group. Results are mean ± SD from three independent experiments, ns, no statistical significance, **p ≤ 0.01, PI, primary infection; SI, secondary infection.

Shortly after reinfection, large numbers of immune cells rapidly accumulated in the cecum when compared with results from the naïve and primary infected mice (**Figure 2A**). By multi-parameter immunohistochemistry, we found that cecum infiltrations by CD4^+^ and CD8^+^ T cells were predominantly restricted to SI mice(**Figure 2B**). In separate quantification of cecal interepithelial lymphocytes (IEL) and lamina propria lymphocytes (LPL), the percentage of CD4^+^ and CD8^+^ T lymphocytes in the IEL and total CD4^+^ T cells in the LPL were similar for the three groups of mice (**Figures 2C, D**). However, the proportion of CD8^+^ T cells in the lamina propria of SI mice was elevated: 13.8% in PI versus 22.7% in SI mice (**Figures 2C, D**). Our current observations showed that significant elevation of CD8^+^ T cells in the lamina propria of the cecum after reinfection with *E. falciformis*.

### Increased CD8^+^ T cells in cecal lamina propria after reinfection were mainly CD8^+^ Trm subpopulation

**Figure 3:**
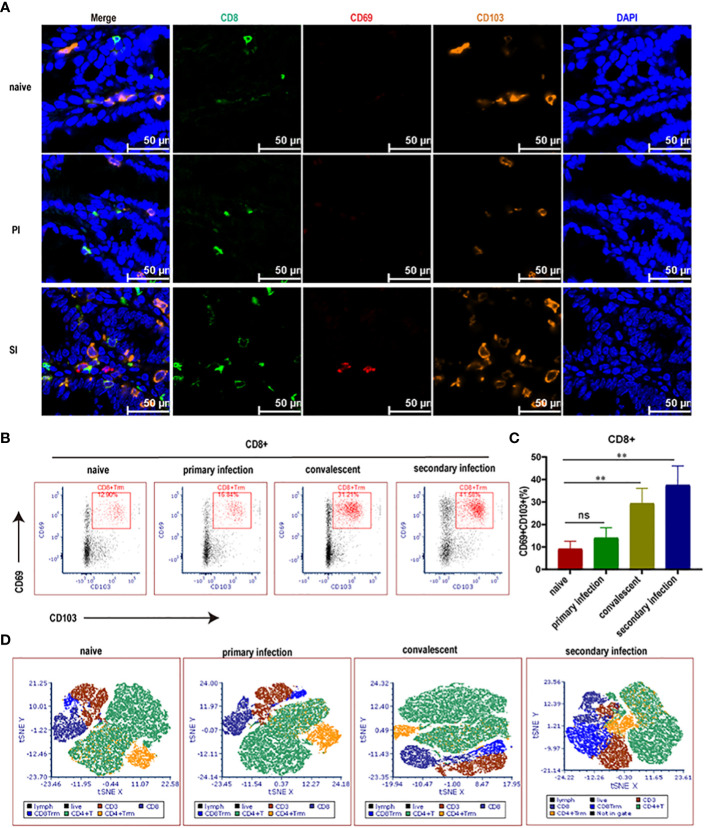
Cecal CD8^+^ Trm cells (in blue color) rose rapidly in reinfected mice. **(A)** Multi-parameter immunohistochemical staining for CD8^+^, CD69^+^ and CD103^+^ Trm cells enriched in the cecum at 24 hours post primary infection and reinfection with 5000 *E. falciformis*. *n*=3 per treatment group, scale bar =50 μm. **(B)** Representative flow cytometric plots for expression of CD69*^+^* and CD103*^+^* on CD8^+^ T cells in LP of cecum at 24 hours post primary infection and reinfection with 5000 *E. falciformis*. *n*=6 per treatment group. **(C)** Summary bar graph of CD8^+^Trm, gated on live CD8^+^ cells. **(D)** t-Distributed Stochastic Neighbor Embedding (tSNE) plots of LP-infiltrating single CD3^+^ cells. Results are mean ± SD from three independent experiments, ns, no statistical significance, **p ≤ 0.01.

Based on multi-parameter immunohistochemical staining, CD8^+^ T cells in SI mice were mostly Trm subsets (**Figure 3A**). The proportion of CD8^+^ Trm to total CD8^+^ T cells in LPL was much higher in cecal lamina propria of SI mice (40.9%) when compared with naïve (9.1%) and PI mice (14.1%) (**Figures 3B, C**). Other subsets of CD8 cells, CD8^+^CD62L^-^CD69^-^ (Tem) and CD8^+^CD62L^+^CD69^-^ (Tcm and naïve T), accounted for 13.9% and 22.2% of the total CD8^+^ T cells, respectively, in cecal lamina propria of SI mice (**Supplementary Figure 1**). Further, T-SNE analysis using flow cytometry data also revealed that the elevated CD8^+^ T cells after reinfection were mainly the CD8^+^ Trm subpopulation (**Figure 3D**, **Supplementary Figure 2**), suggesting that cecum-resident memory CD8^+^ T cells expanded rapidly after reinfection with *E. falciformis*.

### Cecal CD8^+^ Trm cells produced high levels of pro-inflammatory cytokines and cytotoxic effector molecules after *E. falciformis* reinfection

**Figure 4:**
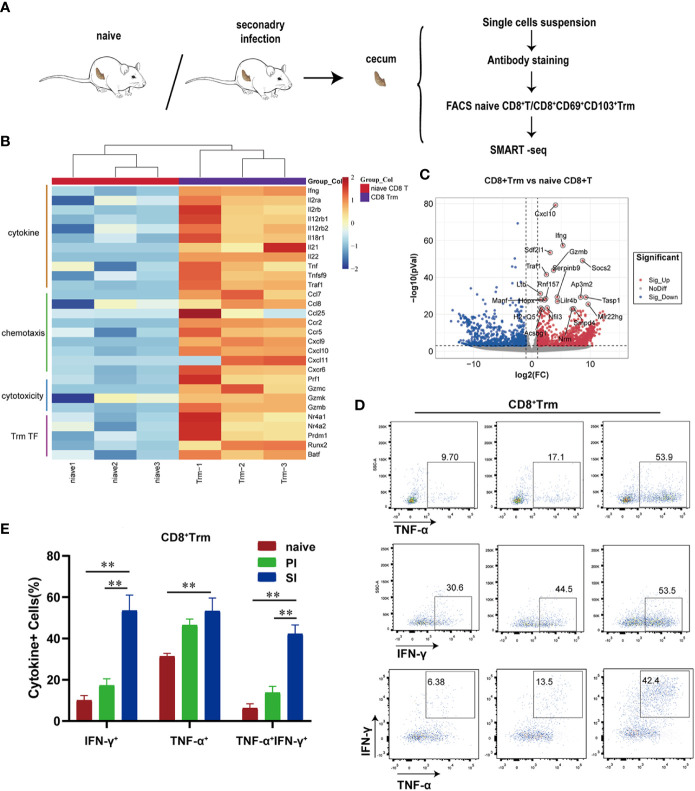
Cecal CD8^+^ Trm cells produced high levels of pro-inflammatory cytokines and cytotoxic effectors molecules after *E. falciformis* reinfection. **(A)** Schematic of SMART-seq experiments. CD8^+^ T-cells were obtained from cecal lamina propria of naïve and reinfected mice (24 hours post reinfection) for comparison. **(B)** Heatmap of selected differentially expressed genes in two groups (>2 fold; *p* < 0.05). **(C)** Volcano plots selected differentially expressed genes between naïve CD8^+^ T and CD8^+^ Trm cells. **(D)** Flow cytometry plots representing IFN-γ and/or TNF-α production by cecal CD8^+^ Trm cells. *n*=6 per treatment group. **(E)** Summary bar graph of IFN-γ and/or TNF-α producing CD8^+^ Trm cells in LP, **p ≤ 0.01, PI, primary infection; SI, secondary infection.

Next, to more comprehensively characterize the phenotype of CD8^+^ Trm cells, we used cell sorting technology to enrich CD8^+^CD69^+^CD103^+^ Trm and naïve CD8^+^ T cells for deep-sequencing (SMART-seq2) (**Figure 4A**), with a focus on correlation heatmaps (**Supplementary Figure 3A**). Highly reproducible results demonstrated that the CD8^+^ Trm cells expressed high levels of genes involved in chemotaxis (e.g., *Ccl7*, *Ccl8*, *Ccl25*, *Ccr2*, *Ccr5*, *Cxcl9*, *Cxcl10*, *Cxcl11*, *Cxcr6*) or encoding cytotoxic molecules (*Prf1*, *Gzmc*, *Gzmk*, *Gzmb*), transcription factors related to tissue residence (*Runx2*, *Nr4a1*, *Nr4a2*, *Prdm1*, *Batf*) and effector cytokines (e.g., *Ifng* and *Tnf*), cell proliferation and other defense mechanisms (*Il2ra*, *Il2rb*, *Il12rb*, *Il12rb2*, *Il18r1*, *Il21*, *Il22*) (**Figures 4B, C**, **Supplementary Figure 3B**). In GO enrichment analysis, these genes were mainly clustered with pathways responsible for lymphocyte chemotaxis, proliferation, adhesion and cytokine production (**Supplementary Figure 3C**). A PPI network identified 29 nodes and 168 edges (**Supplementary Figure 3D**), whereas GSEA enrichment analysis confirmed a cytokine/chemokine-mediated pathway as part of an adaptive immune response (**Supplementary Figure 3E**). Intracellular cytokine staining assays further revealed that IFN-γ and/or TNF-α-positive CD8^+^ Trm cells were detected at a higher proportion in cecal lamina propria of SI mice when compared with naïve and PI mice (**Figures 4D,E**). Thus, CD8^+^ Trm cells in SI mice were featured by both pro-inflammatory cytokines and cytotoxic effector molecules.

### CD8^+^ Trm cells were responsible for direct protection against *E. falciformis* reinfection

**Figure 5:**
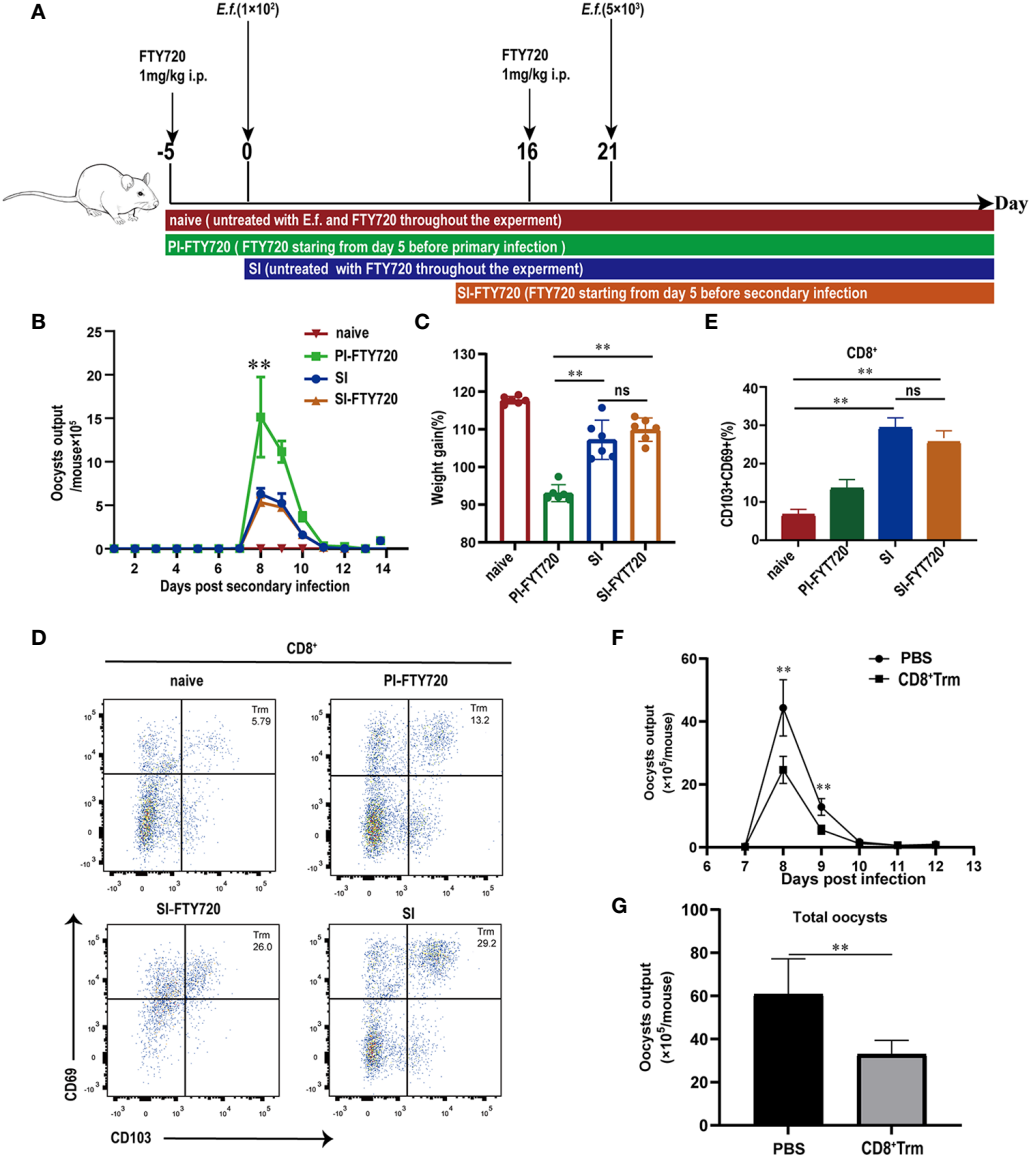
CD8^+^ Trm cells were responsible for protection against *E. falciformis* infection. **(A)** Experimental design for treatment with FTY720 scheme. Mice were given FTY720 by intraperitoneal injection for 5 d before primary or secondary infection and during infection with *E. falciformis*. **(B)** Kinetics of oocyst output of mice treated or untreated with FTY720 at 6-13 days post reinfection with 5000 *E. falciformis*. *n*=6 per treatment group. **(C)** Body weight change of mice treated or untreated with FTY720 at 8 days post reinfection with 5000 *E. falciformis*. *n*=6 per treatment group. **(D)** Representative flow cytometric plots for expression of CD69 and CD103 on CD8^+^ T-cells in LPL from mice treated or untreated with FTY720 at 24 hours post reinfection with 5000 *E. falciformis*. *n*=6 per treatment group. **(E)** Summary bar graph of CD8^+^ Trm, gated on live CD8^+^ cells. **(F)** Kinetics of oocyst output of mice received CD8^+^ Trm or PBS at 6-13 days post infection with 100 *E.falciformis*. *n*=5 per treatment group. **(G)** Total oocyst output of mice received CD8^+^ Trm or PBS. *n*=5 per treatment group. Results are mean ± SD from three independent experiments, ns, no statistical significance between treatment groups, **p ≤ 0.01, PI, primary infection; SI, secondary infection.

We next investigated the ability of CD8^+^ Trm cells to directly confer immunity to *E. falciformis* infection (**Figure 5A**). Two groups of mice that started with continuous FTY720 treatment either on day 5 prior to primary infection or on day 5 before reinfection were compared with naïve and SI mice without FTY720 treatment. In contrast to the exacerbating effect of FTY720 on a primary infection (**Supplementary Figure 4**), treatment of mice prior to reinfection did not alter the course of a secondary infection. The group, treated with FTY720 starting from day 5 before primary infection, had the worst outcomes, as reflected by high oocyst output, low body weight, and low CD8^+^ Trm cell counts (**Figures 5B–D**), but these outcome measures were quite similar in the SI and SI-FTY720 groups regardless of FTY720 treatment. Flow cytometric analysis did confirm that FTY720 treatment impaired circulation of lymphocytes (CD3^+^, CD4^+^ and CD8^+^ T cells), resulting in a lower number of T cells in peripheral blood (**Supplementary Figure 5**), but CD8^+^ Trm cells in the cecum of SI-FTY720 mice (at 24 h after reinfection) was not affected (**Figure 5E**). When CD8^+^ Trm cells from cecum of reinfected mice were adoptively transferred to naïve mice, those challenged 1 d after CD8^+^ Trm transfer had significantly lower *E. falciformis* shedding than control mice that received sham (PBS) injections (**Figures 5F, G**). Collectively, these findings demonstrated that the CD8^+^ Trm cells that develop during the primary infection and reside locally in the cecum are responsible for protective immune response against secondary infection.

## Discussion

Consistent with our hypothesis, protective immunity to *Eimeria* reinfection, is mediated by tissue-resident CD8^+^ T cells with the Trm phenotypes. These cells are readily induced by low-dose *E. falciformis* infection, as is the case of live vaccination, and they proliferate locally and rapidly after reinfection to limit parasite burden. Thus, previous notions that CD8^+^ T cells are essential to long-lasting immune protection against *Eimeria* reinfection (12, 13) can be attributed to intestinal tissue-resident CD8^+^ T cells. Evidently, the development and maturation of schizonts in the intestine of reinfected mice are affected mainly at the stage of first generation (early) schizonts. Thus, suppression of first generation schizonts development appears to be the most critical function of memory CD8^+^ T responses against *Eimeria* infections (14, 15), as reported earlier in mice infected with *E. vermiformis* (16) and in chickens infected with *E. tenella* (17). Naïve CD8^+^ T cell responses to infections need several days to reach a robust level, which leaves the invading parasites ample time to proliferate and mature (18, 19). In contrast, CD8^+^ Trm cells respond within hours when triggered by a secondary encounter to offer prompt immune protection (20).

Memory T cells are known to persist at various tissue sites for months (6). For Trm cells that do not recirculate (18), their contribution to local immune protection (21, 22), including intestine (23), brain (24), skin (25) and lung (26), is rapid and effective (27). Of note, in convalescent mice, treatment with anti-CD4 or anti-CD8 monoclonal antibodies has little effect on pre-existing immunity to reinfection with *E. vermiformis* (13). The explanation may be that intestine-resident memory cells, which confer superior protection relative to peripheral Tem cells and Tcm cells (28), are not susceptible to depletion by intravenous injection of corresponding antibodies, and their mobilization is already sufficient in mounting an effective immune protection. Indeed, our study suggests that even after a chemical blockade to the recruitment of peripheral T cells in convalescent mice (using the FTY720), control of *E. falciformis* reinfection in the SI-FTY720 group is still intact when CD8^+^ Trm cells are not affected. In coccidiosis adoptive transfer has previously been described in rats, mice (12, 16, 29, 30) and chickens (31). The general point of all these reports is that they necessitated the injection of very large numbers of cells (>10^7^ or >10^8^) to reduce oocyst production in recipients by at least 50%. When the protective properties of spleen and MLN cells were examined separately, it became clear that a large number of spleen cells were required, probably because the cells used were not directly associated with the gut, the number of MLN (gut associated) cells required to transfer immunity to this infection was not unduly large. In this paper we observed that transfer of 10^6^ CD8^+^ Trm cells provided similar protection as transfer of 10^7^ MLN CD8^+^ T (12). This result indicated that CD8^+^ Trm has very effective protective effect against *Eimeria* infection.

Immune protection attributed to Trm cells is characterized by cytokines and chemokines that amplify local recruitment of other innate and adaptive immune cells (32, 33). In our model system, CD8^+^ Trm cells express genes encoding chemotaxis (*Ccl7*, *Ccl8*, *Ccl25*, *Ccr2*, *Ccr5*, *Cxcl9*, *Cxcl10*, *Cxcl11*, *Cxcr6*) and pro-inflammatory cytokines, as reported by others (34). Trm patrol the gut microenvironment and join the first wave of sentinels during recall responses, with rapid proliferation and function upon encountering their cognate antigen or pathogen (35, 36), which are also consistent with our findings on IFNγ and TNF (37, 38), as well as other effector molecules (*Gzmb*, *Gzmc*, *Gzmk* and *Prf1*) that dictate cytotoxic activities. These and other molecular signatures associated with CD8^+^ Trm provide reassuring evidence that the intestinal CMI mechanisms are complex and highly coordinated (39–42). It remains challenging to manipulate such responses through the use of subunit or DNA vaccines, as the induction of mucosal immunity in the gut has attracted little attention in ongoing vaccine development. Our findings explained a protective mechanism of live-oocyst based anti-*Eimeria* vaccines, which may serve as an important directive for developing effective vaccines against medically important apicomplexan diseases.

## Data availability statement

The datasets presented in this study can be found in online repositories. The names of the repository/repositories and accession number(s) can be found below:PRJNA863079 (SRA).

## Ethics statement

The animal study was reviewed and approved by China Agricultural University Institutional Animal Care and Use Committee guidelines.

## Author contributions

XS and FS conceived and designed this study and analyzed the data. FS carried out the experiments and drafted the manuscripts. SZ, NZ, YY, PS, and XT contributed to help the statistical analysis and help to draft the manuscripts. XS and XL supervised the study implementation and revised the manuscript. All authors contributed to the article and approved the submitted version.
