# Supplementary material for: Tissue-resident, memory CD8+ T cells are effective in clearing intestinal Eimeria falciformis reinfection in mice
Source: Front Immunol. 2023 Feb 14;14:1128637. doi: 10.3389/fimmu.2023.1128637 (PMC9971219; doi:10.3389/fimmu.2023.1128637)
Supplement: Supplementary file 1 [file Image_1.pdf]

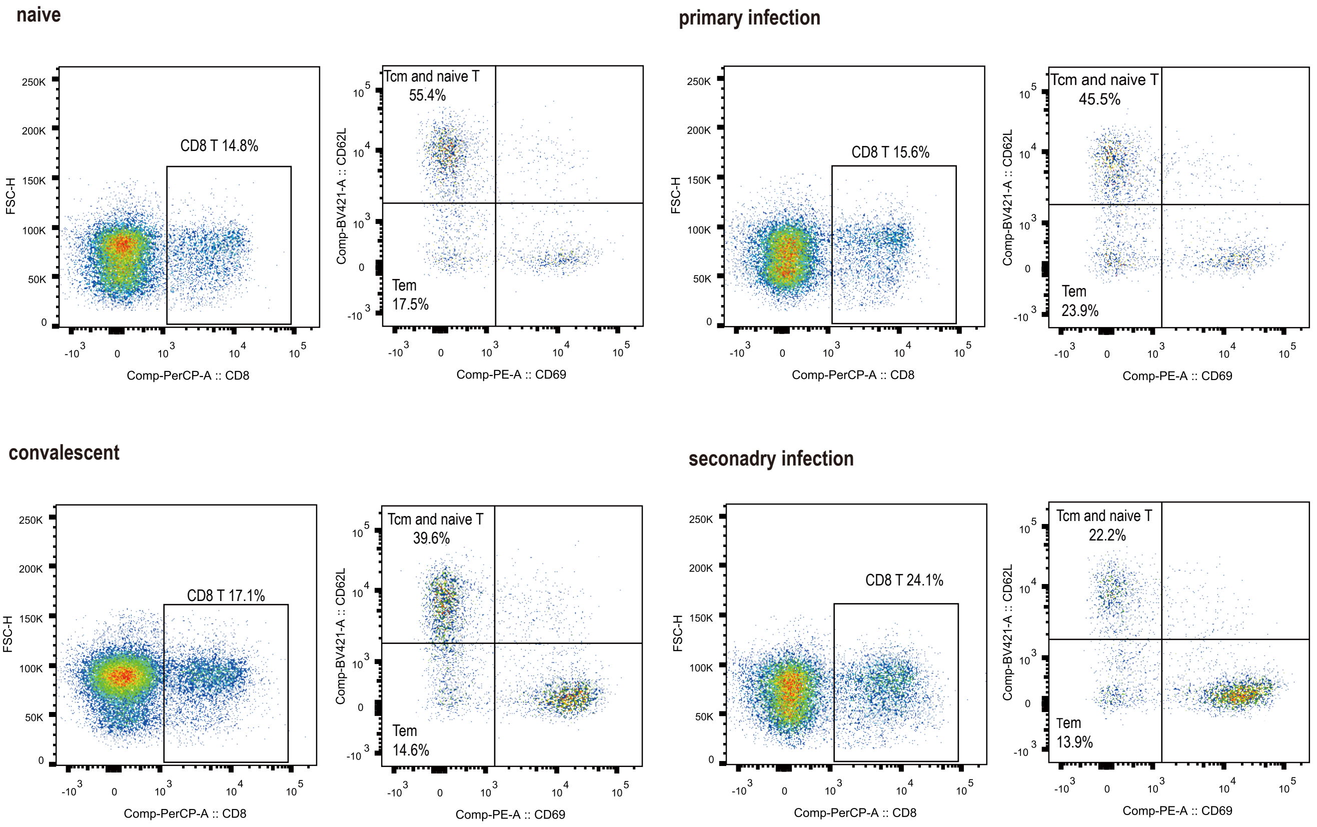

Supplementary Fig. 1 CD8$^+$ Tem, CD8$^+$ Tcm and naïve T cells in LPL of cecum from naïve, primary infected, convalescent and reinfected mice. Representative flow cytometric plots for expression of CD69 and CD62L on CD8$^+$ T cells in LPL from naive mice (A), singly infected mice (B), convalescent mice (C) and reinfected mice (D) at 24 hours post infection and reinfection with 5000 oocysts of E. falciformis (n=6 per group). Results are representative of two independent experiments.
