# Supplementary material for: Tissue-resident, memory CD8+ T cells are effective in clearing intestinal Eimeria falciformis reinfection in mice
Source: Front Immunol. 2023 Feb 14;14:1128637. doi: 10.3389/fimmu.2023.1128637 (PMC9971219; doi:10.3389/fimmu.2023.1128637)
Supplement: Supplementary file 2 [file Image_2.pdf]

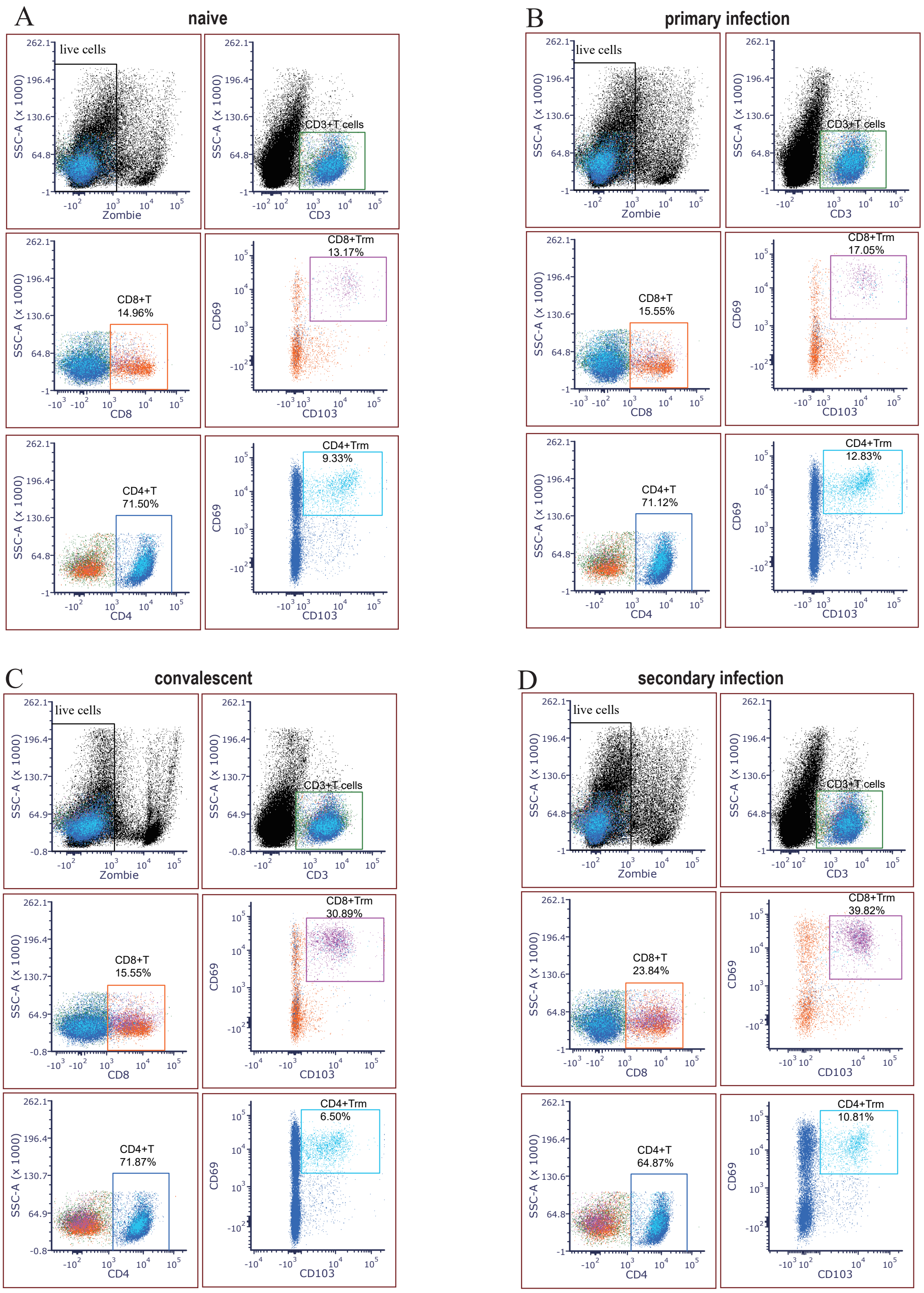

Supplementary Fig. 2 CD4^{+} T, CD4^{+} Trm, CD8^{+} T and CD8^{+} Trm cells in LPL of cecum from naïve, primary infected, convalescent and reinfected mice. Representative flow cytometric plots for expression of CD69 and CD103 on CD8+ or CD4+ T-cells in LPL from naïve mice (A), singly infected mice (B), convalescent mice (C) and reinfected mice (D) at 24 hours post infection and reinfection with 5000 E. falciformis (n=6 per group). Results are representative of two independent experiments.
