# Supplementary material for: Tissue-resident, memory CD8+ T cells are effective in clearing intestinal Eimeria falciformis reinfection in mice
Source: Front Immunol. 2023 Feb 14;14:1128637. doi: 10.3389/fimmu.2023.1128637 (PMC9971219; doi:10.3389/fimmu.2023.1128637)
Supplement: Supplementary file 3 [file Image_3.pdf]

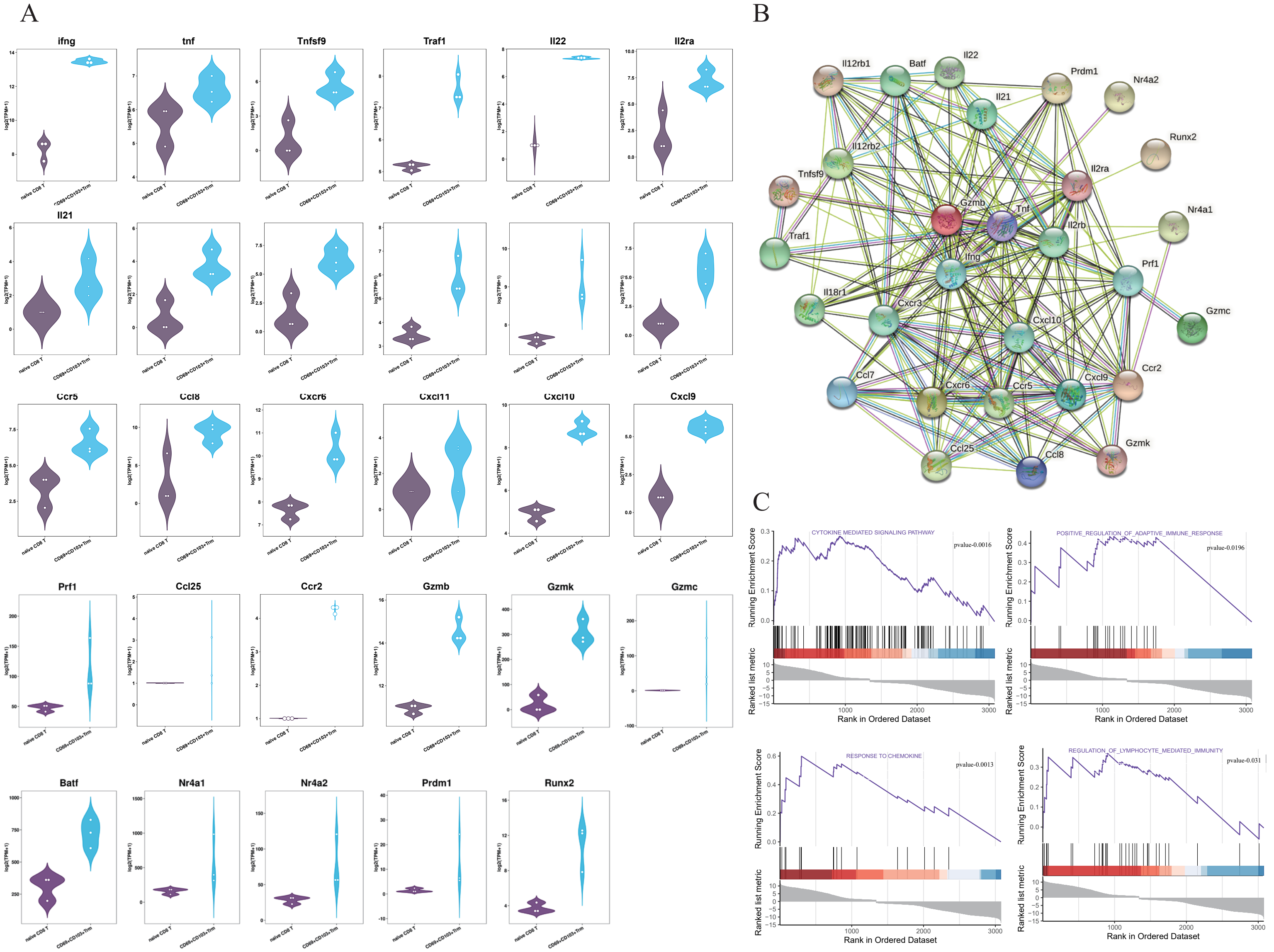

Supplementary Fig. 3. Differential analysis of gene expression profiles of CD8^{+} Trm versus naïve CD8^{+} T cells. (A) Violin plot of selected differentially expressed genes. (B) Protein-protein interaction (PPI) network of selected differentially expressed genes. (C) GSEA enrichment analysis of the differentially expressed gene.
