# Supplementary material for: Tissue-resident, memory CD8+ T cells are effective in clearing intestinal Eimeria falciformis reinfection in mice
Source: Front Immunol. 2023 Feb 14;14:1128637. doi: 10.3389/fimmu.2023.1128637 (PMC9971219; doi:10.3389/fimmu.2023.1128637)
Supplement: Supplementary file 4 [file Image_4.pdf]

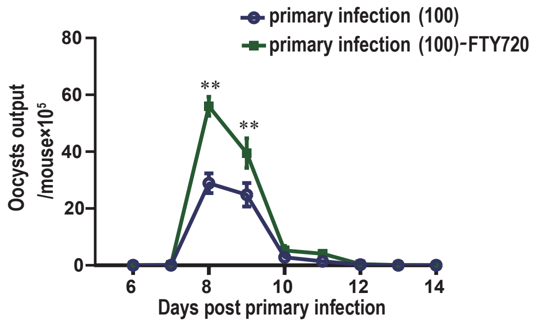

Supplementary Fig. 4 Kinetics of oocyst output of mice treated and untreated with FTY 720 in primary infection with 100 E. falciformis. six mice per group at each time point. Results are mean ± SD from two independent experiments, *$p \leq 0.05$, **$p \leq 0.01$.
