# Supplementary material for: Tissue-resident, memory CD8+ T cells are effective in clearing intestinal Eimeria falciformis reinfection in mice
Source: Front Immunol. 2023 Feb 14;14:1128637. doi: 10.3389/fimmu.2023.1128637 (PMC9971219; doi:10.3389/fimmu.2023.1128637)
Supplement: Supplementary file 5 [file Image_5.pdf]

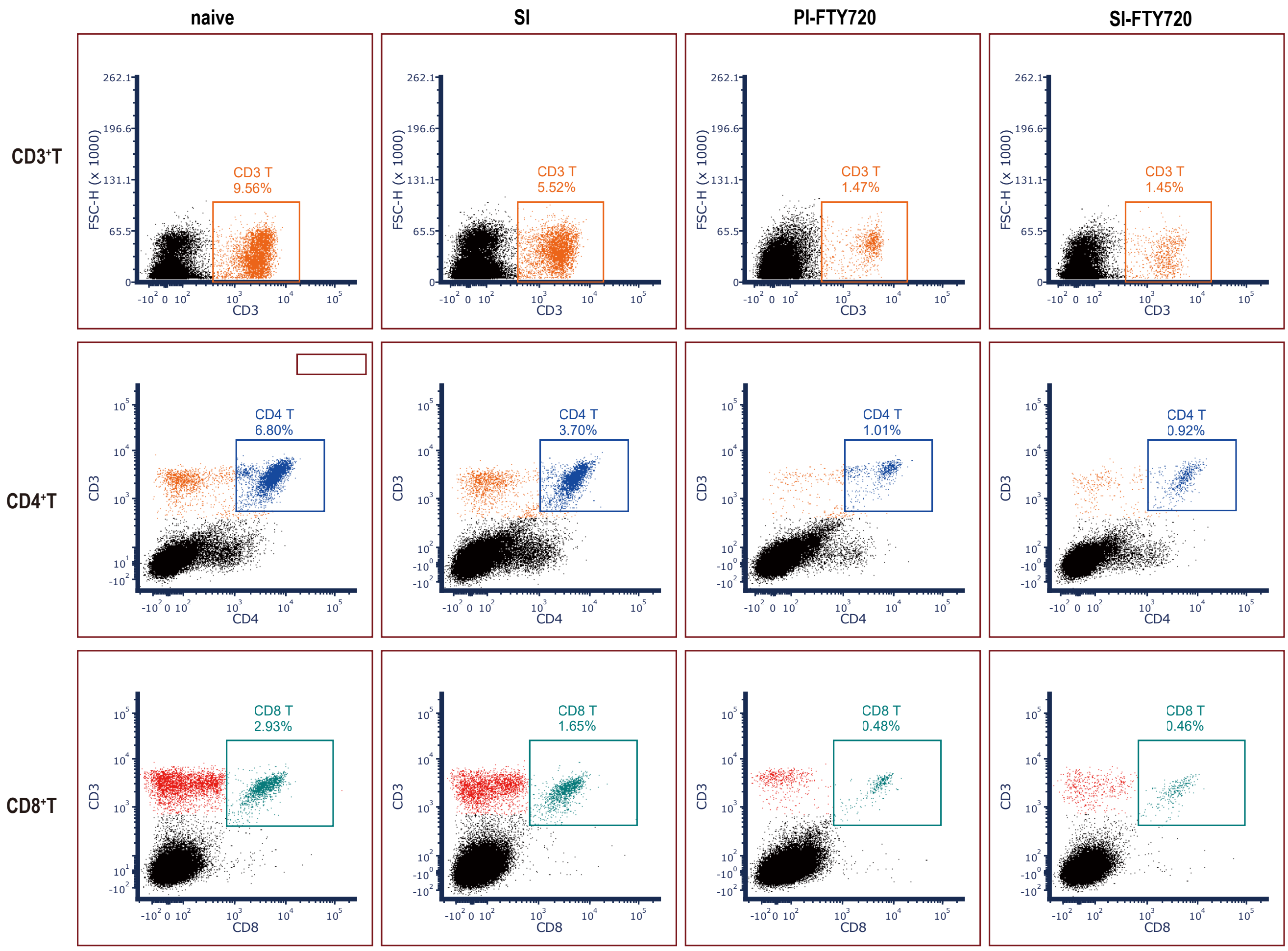

Supplementary Fig. 5 Representative flow plots of CD3+, CD4+ and CD8+ T-lymphocytes in peripheral blood of mice treated and untreated with FTY720. PI= primary infection, SI= secondary infection.
